# Relative efficacy of different types of exercise for treatment of knee and hip osteoarthritis: protocol for network meta-analysis of randomised controlled trials

**DOI:** 10.1186/s13643-016-0321-6

**Published:** 2016-09-02

**Authors:** Siew-Li Goh, Monica S. M. Persson, Archan Bhattacharya, Michelle Hall, Michael Doherty, Weiya Zhang

**Affiliations:** 1 Academic Rheumatology, Division of Rheumatology, Orthopaedics and Dermatology, School of Medicine, University of Nottingham, Nottingham, UK; 2 Sports Medicine Unit, University of Malaya, Kuala Lumpur, Malaysia; 3 Arthritis Research UK Centre for Sports, Exercise and Osteoarthritis, University of Nottingham, Nottingham, UK; 4 Arthritis Research UK Pain Centre, University of Nottingham, Nottingham, UK; 5 Division of Physiotherapy and Rehabilitation Sciences, University of Nottingham, Nottingham, UK; 6 Academic Rheumatology, Clinical Sciences Building, Nottingham City Hospital, Hucknall Road, Nottingham, NG5 1PB UK

**Keywords:** Osteoarthritis, Lower limb, Exercise, Network meta-analysis, RCT, Hip, Knee, Pain

## Abstract

**Background:**

‘Exercise’ is universally recommended as a core treatment for knee and hip osteoarthritis (OA). However, there are very few head-to-head comparative trials to determine the relative efficacy between different types of exercise. The aim of this study is to benchmark different types of exercises against each other through the use of a common comparator in a network meta-analysis of randomised controlled trials (RCTs).

**Methods:**

This study will include only RCTs published in peer-reviewed journals. A systematic search will be conducted in several electronic databases and other relevant online resources. No limitations are imposed on language or publication date. Participants must be explicitly identified by authors as having OA. Interventions that involved exercise or comparators in any form will be included. Pain is the primary outcome of interest; secondary outcomes will include function and quality of life measures. Quality assessment of studies will be based on the modified Cochrane’s risk of bias assessment tool. At least two investigators will be involved throughout all stages of screening and data acquisition. Conflicts will be resolved through discussion. Conventional meta-analysis will be performed based on random effects model and network meta-analysis on a Bayesian model. Subgroup analysis will also be conducted based on study, patient and disease characteristics.

**Discussion:**

This study will provide for the first time comprehensive research evidence for the relative efficacy of different exercise regimens for treatment of OA. We will use network meta-analysis of existing RCT data to answer this question.

**Systematic review registration:**

PROSPERO CRD42016033865

**Electronic supplementary material:**

The online version of this article (doi:10.1186/s13643-016-0321-6) contains supplementary material, which is available to authorized users.

## Background

Osteoarthritis (OA) is the most common form of arthritis, characterised structurally by focal articular cartilage loss, subchondral bone remodelling and changes in the synovium, capsule and periarticular tissues [1]. The aetiology of OA is multifactorial, and its prevalence is higher in the elderly and in women [2]. The knee joint appears to be more commonly affected than the hip [2]. It is a major health burden and has been ranked 11th out of all common causes of disability globally [3]. Patients with symptomatic OA may experience substantial reduction in quality of life (QoL), limitation in mobility and higher mortality and morbidity [4, 5].

In the absence of any definitive cure [6, 7], non-pharmacological therapy with adjunctive pharmacological analgesic use are the mainstay of management in knee and hip OA, with joint replacement surgery being reserved for severe disease that is resistant to conservative management [8, 9]. Pain-related limitation in physical activity and lower limb muscle weakness are common problems in knee and hip OA but are potentially reversible. Hence, exercise therapy is one of the core non-pharmacological therapies universally recommended for all patients with OA. Exercise therapy is as effective as pharmacological agents in providing symptom relief and functional improvement, but without the serious side effects associated with systemic analgesics [10].

### Description of the intervention

Physical activity is defined as body movement that is produced by the contraction of the skeletal muscles and that increases energy expenditure, whereas exercise is planned, structured and repetitive movement to improve or maintain one or more components of physical fitness [11]. Exercise has proven benefits in the various components of health-related physical fitness—defined by the American College of Sports Medicine (ACSM) to consist of cardiorespiratory fitness, body composition, muscular strength, muscular endurance and flexibility [12].

Exercise programmes are complex interventions that vary with exercise type, frequency, intensity and duration, as well as the mode of delivery (individual-group, supervised-unsupervised or facility-home-based). There is a common tendency to pool clinical trial data on exercise as an indistinct entity when formulating conclusions on exercise effects [13]. Such results can be misleading because the effects of exercise can be moderated by the nature of the exercise programmes itself and by both patient and disease characteristics [14, 15].

Most exercise interventions for OA conventionally fall into one of the following physical performance categories: strengthening, aerobic, flexibility and skills/balance. In theory, the health benefits accrued are specific to the type of exercise. For example, aerobic activity to improve cardiorespiratory fitness can improve sleep and well-being and reduce all-cause mortality, whereas strengthening primarily improves local muscle function and proprioception to improve joint stability and local biomechanical functioning. However, there is evidence that both forms of exercise can reduce pain and improve function so both are recommended in most recent guidelines [8, 11, 16].

Other than strengthening or aerobic exercises, range of motion (ROM) exercise is also believed to be beneficial in improving symptoms and function. This is especially useful when functional and structural properties of periarticular soft tissue have been compromised following acute knee swelling or prolonged joint immobilisation [17]. Other types of exercise that incorporates ‘mind and body’ components such as Tai Chi and Yoga are also gaining interest for its role in improving symptoms and function [18]. It is possible that these may have additional benefits such as modulation of the inflammatory response [19] and reduction of central sensitisation in people with OA [20].

As a result, an increasing number of exercise options are being recommended [8–10]. However, relative efficacy of different exercises (in terms of pain reduction, improvement of function and quality of life) still remains largely unknown. More interestingly, whether different types of exercise can be tailored to individual patient characteristics to maximise the benefits of exercise therapy has yet to be investigated.

### Importance of the project

Considering the complexity of trial designs involving exercise, it is unlikely that primary research can satisfactorily provide a good overview relating to the efficacy of each individual exercise—at least not without incurring a huge demand in resources in order to perform multiple head to head comparison trials. For these reasons, the comparisons between the effects of various exercises may be examined more efficiently through a network meta-analysis (NMA) using existing clinical trial data.

NMA is a method which attempts to estimate the difference between two treatments (A and B) through a common comparator (C) when the direct evidence between these two treatments is not available [21]. There may be several common comparators (nodes) shared by different treatments at different levels. When common comparators (nodes) are identified, treatments from different trials can be networked. We will then be able to estimate the relative effects of the different treatments (exercises in this case) against each other through these common comparators. In other words, the effects of different types of exercises in improving symptoms, function and quality of life of patients with knee and hip OA can be determined by integrating evidence from existing comparison trials.

## Methods

This protocol is prepared conforming to Preferred Reporting Items for Systematic Reviews and Meta-Analyses Protocols (PRISMA-P) as closely as possible (Additional file 1).

### Criteria for considering studies for this review

#### Types of studies

Randomised controlled trials (RCTs) (including cross-over and cluster randomised trials) which examine the effects of exercise interventions in adults with knee or hip OA in all settings will be included. Studies will be considered as RCT if authors had explicitly stated that it is randomised [22] or when randomisation cannot be ruled out. Quasi randomised trials are excluded.

#### Participants

People with symptomatic OA of the knee and hip joint, diagnosed clinically (e.g. American College of Rheumatology Criteria), or radiographically (e.g. Kellgren Lawrence grading), or by the use of other imaging (e.g. magnetic resonance imaging) will be included. There is no restriction on the severity or stage of the disease. Hence, studies involving patients from early to ‘end-stage’ OA, including participants with pain following joint replacement, will be considered.

Studies using mixed disease categories from which the subgroup of knee and/or hip OA cannot be identified or fail to explicitly specify the joint condition as OA will be excluded. Arbitrarily, >80 % of joint replacement patients need to have their surgery attributed to OA in order for the study to be included [23].

#### Interventions

Interventions that involve exercise prescribed in any form, such as strengthening exercise, aerobic exercise or mind-body exercise (e.g. tai-chi, yoga), will be eligible for inclusion. In instances where the term ‘exercise’ is not used by the investigators, any physical training that fulfils the basic characteristics of exercise (i.e. a structured programme which is repeated/practised on a regular basis, e.g. three times a week) will also be included. Single and combined interventions will be included, for example exercise, ±adjunct treatment versus a non-exercise intervention such as education, manual therapy or electrotherapy. Additional file 2: Table S1 shows how the pairwise comparisons from a study will determine its eligibility.

Exercise will be classified based on four core exercise types described by American College of Sports Medicine (ACSM) [24]. An additional category of mind-body exercise will also be included in our study. The classifications are as follow:

- Strengthening/resistance exercise: exercise that aims to improve the muscle’s ability to exert force and involves applying resistance against a contracting muscle.
- Aerobic exercise: exercise that aims to improve cardiorespiratory fitness and involves repetitive movement of large muscle groups, performed at moderate to vigorous intensity for prolonged periods of time.
- Flexibility exercise: exercise that improves the ability to move a joint throughout its range of motion and includes various types of stretching exercises.
- Neuromotor exercise: exercise that improves motor skills such as balance and coordination.
- Mind-body exercise: exercise that combines a physical exercise with meditation or mindfulness. The latter is defined as the intention to be aware and engaged in the present moment, i.e. attention on your breath and movements without disturbed by other issues [25]. It is a set of mindful movements with a primary purpose of relaxation. The prototype of this exercise includes Tai Chi, Qi Chong and Yoga.

If the authors had not clearly identified the exercise element of interest, intervention that includes more than one category of exercise will be categorised as mixed exercise. Other components (such as home based versus class based) will be added to each if necessary to flag out the difference. Further classifications may be made as appropriate. SLG (a sports physician) and MH (a physiotherapist) will be involved in classifying the exercise interventions.

#### Comparators

All comparators which have been used in the exercise trials will be included. However, for the purpose of the network meta-analysis, we need to include a common comparator across different exercises. The common comparator is defined as the comparator which had been used by at least two trials for two different exercises. These could be a no treatment/attention control, a waiting list control, a leaflet/education control, a ‘sham’ exercise or an active control (another type of exercise or intervention).

#### Outcomes

The primary outcome will be pain. Secondary outcomes will include self-reported function, objective measured function/physical test and QoL. Except for outcomes based on physical testing, a hierarchical selection of measurement scale following Fransen [26] and Regnaux [27] will be adopted if more than one scale is being used for the same outcome. However, a lower ranked scale may be given priority over a higher ranked scale if (i) it is more comprehensively reported in the study or (ii) if the direction of effect of the higher ranked scale is unclear.

- Primary outcome—pain
  The hierarchical selection, in descending order of preference, is as follows:
  Pain overall
  Pain on walking
  Western Ontario and McMaster Universities Osteoarthritis Index (WOMAC) pain subscale
  Pain on activities other than walking
  WOMAC global scale
  Lequesne osteoarthritis (OA) index global score
  Other algo-functional scale
  Patient’s global assessment
  Other outcomes
- Secondary outcomes
  (i) Functional outcomes—self-reported and objective outcomes
    Hierarchical selection for self-reported measures in descending order of preference is as follows:
    Global disability score
    Walking disability
    WOMAC disability sub-score
    Composite disability scores other than WOMAC
    Disability other than walking
    WOMAC global scale
    Lesquesne OA index global score
    Other functional scales
    For objective performance-based outcomes, a different consideration is used and may involve identification of the commonest physical test through ‘vote counting’—i.e. the test that is reported by most studies. This is because no physical tests, either singly or in combination, have been deemed to be sufficiently investigated or robust to warrant any definitive recommendations for the assessment of physical function in hip and knee OA patients [28]. Alternatively, if the correlations between the tests are known, we may consider pooling the results and generate only one effect size estimate. In the event of any discrepancies, a consensus will be sought within the research team.
  (ii) QoL
    Hierarchical selection for QoL measures is as follows:
    General health of short form (SF)-36 or SF-12
    EuroQol (EQ)-5D
    World Health Organization quality of life
    Mental/physical component summary of short form (SF)-36
    Mental/physical component summary of short form-12
    Sickness impact profile
    Nottingham health profile
    Other qualities of life scales
    Others
  (iii) Others
    If widely reported by authors, additional measurements such as structural, biomechanical or physiological parameters, including features on imaging (radiographs, magnetic resonance imaging, ultrasound), will also be included.

#### Study time points

There is no pre-specified study endpoint for eligibility. Outcomes at different time points during the follow-up period will be recorded, and the commonest point across all trials will be used as a primary outcome point. Additionally, outcome points during follow-up will be grouped into intervals (e.g. ≤1 month, 1–3 months, 3–6 months, 6–12 months, 12–24 months etc.) for a secondary analysis for the time-dependent effect.

### Search methods for identification of studies

#### Search strategy

This meta-analysis aims to include all studies for exercise therapy in OA published in peer-reviewed journals. The following electronic bibliographic databases will be systematically searched from their dates of inception: Allied and Complementary Medicine Database (AMED), The Cochrane Central Register of Controlled Trials (CENTRAL), Cumulative Index to Nursing and Allied Health Literature (CINAHL), Excerpta Medica Database (EMBASE), MEDLINE, Physiotherapy Evidence Database (PEDro), PubMed, SPORTDiscus and Google Scholar. There will be no restrictions on languages. An example of MEDLINE search is shown in Additional file 3.

The reference lists of systematic review protocols published in Cochrane Library since 2014 will be used to supplement the electronic database search. Abstracts may be included if sufficient information for data extraction with/without risk of bias assessment is provided. Publications of study protocols will be flagged pending the full publication of the trial.

### Data collection

#### Study selection process

Preliminary screening is by title and abstract. Full text of potential citations will be retrieved for final screening and data extraction. Study selection will be done by one reviewer with the second reviewer performing periodical validations at random. A third reviewer will be involved if any discrepancies arise—i.e. if either one of the reviewer is unsure or could not agree with each other.

#### Data extraction and management

A structured database is created in Microsoft Access for data entry. In situation where studies are able to contribute two pairwise comparisons, data from four intervention groups will be extracted, and the study will be considered as two separate comparisons. Conversely, studies that have produced companion publications may be combined to maximise the yield of data.

When pairing of multiple groups cannot be performed satisfactorily (such as when there are three groups in a study), then combining groups may be considered. Supposing that all intervention groups are eligible for inclusion, exercise groups may be combined as a single group to be compared against a single control group. The reverse will also be done if two control groups are eligible but only one exercise group is available to contribute to the comparison. Calculation of the standard deviation for the composite group will be performed as described by Cochrane Handbook [29] (Additional file 4). If there is no clinical or practical justification to combine the groups as described, the intervention or the comparator that is deemed to be the simplest/commonest will be chosen instead. The decision for adopting either approach will be described and explained in the table of summary.

The following data will be extracted.

i. Study level include:
  Study identification details (i.e. title, author, year of publication)
  Number of study arms
  Number of patients
  Duration of study
  Population: hospital/healthcare centre, community
  Study design: parallel, crossover
ii. Participant level:
  Demographic: age, gender
  Associated risk factors: body mass index (BMI), history of injury
  Diagnostic criteria used for recruitment
  Composition of patients with knee and hip OA
  Disease severity
iii. Intervention level
  Types of exercise: strengthening, aerobic, balance/skills, flexibility/ROM, mind-body or mixed,
  Measurement of exercise adherence
  Setting: group/class versus individual, hospital versus home-based
  Compositions of patients with hip and knee OA
  Number of patients randomised
  Patient demographics by group
  Comparator types
  Baseline measurements
iv. Outcome level:
  Types of outcome and measurement tool used
  Are results adjusted
  Intention-to-treat (ITT) analysis
  Number of patients analysed/attrition at various stages of study
  Types of effect size and relevant statistics (standard deviation, confidence interval etc.)
  Primary and all measurement time points

An abridged data extraction form (Additional file 5) will be used for data validation. The data extraction will be performed by one reviewer while the second reviewer will independently perform validation. Discrepancies in data will be resolved through discussion between the two reviewers. A third reviewer may also be involved if needed.

#### Missing data

If the required data cannot be extrapolated from the published article, attempts will be made to contact the authors for additional information. Where this is not possible, we will use statistical imputations as appropriate. Details of imputations and assumptions used will be reported.

As calculation of standard deviation is one of the commonly encountered situations, calculations for transforming other forms of summary data to standard deviation (SD) have been listed in Additional file 4. In studies where insufficient information is provided for calculation, the SD may be substituted with the widest SD obtained from other eligible studies.

### Analysis

Approach in analysis will flow from meta-analysis (MA) to NMA and individual patient data (IPD) if the data are available. The MA aims to confirm/update the efficacy of different exercises, whereas the NMA aims to determine the relative efficacy between different types of exercises. The IPD meta-analysis aims to examine who responds better to a specific exercise—that is, to determine predictors of response. Data will be processed using various softwares such as Microsoft Access, Excel, Stata and WinBUGS/SAS.

#### Unit of analysis

For calculation of effect size, the unit of analysis is the mean (and standard deviation) of each trial. Demographic features of participants that were randomised to each group at the commencement of studies will be described. For the summary of effect, independent pairwise comparisons will be the unit of analysis. This is to say, if a study has four independent arms which can be paired, the study will contribute two effect size estimates to the meta-analysis. If a study has only three independent arms, only one effect size will be synthesise for the summary of effect estimate.

Results of different outcomes and at different time point will be reported separately. Any aggregation of data will be indicated and justified.

#### Measures of treatment effect

The effect size of continuous data (pain, functional outcome and QoL) will be based on standardised means difference, Cohen *d*. Whenever possible, the post-treatment mean score will be used for calculation of effect size. If this is not available, the mean change score will be used instead.

From Cohen *d*, a correction factor will be used to obtain the unbiased effect size (Hedges’ *g*). Unless these effect sizes are available by default in the statistical software, calculations will be based on the equations listed in Additional file 4. Point estimates of effect size will be reported with its 95 % confidence interval for pairwise meta-analysis and 95 % credibility intervals for NMA.

An exercise that is able to deliver minimal clinically important difference (MCID) for the respective outcome of interest (e.g. 30 % improvement in visual analogue score from baseline, 20 % improvement in WOMAC function from baseline) will be deemed as effective [30]. An exercise that is able to deliver a predefined MCID compared to another (e.g. difference of 1 score on numerical rating scale) will be considered as more efficacious.

#### Assessment of publication bias

Empirical methods to assess publication bias are not considered to be better than visual assessment of funnel plots [22]. Hence, we will use funnel plot to investigate for publication bias which can be easily detected by presence of gaps in the plot. This scatter plot of study size against treatment effect will demonstrate a void at the lower left section of the graph if the assumption that studies with small sample size and non-significant effect size tend to go unpublished is true.

#### Assessment of heterogeneity

We will use chi-squared (*χ*^2^*)* test to examine homogeneity where level of significance is set at *p <* 0.1. To quantify the impact of the heterogeneity on the pooled estimates, we will use *I*^2^ statistics. The value of *I*^2^ indicates the magnitude of heterogeneity in the analysis and is interpreted as: 0–40 %—might not be important; 30–60 %—may represent moderate heterogeneity; 50–90 %—may represent substantial heterogeneity; and 75–100 %—considerable heterogeneity [29]. Assessment of similarity and consistency of the estimate will be performed to ensure that the direct and indirect estimates agree. If the direct and indirect evidence agree, the estimates will be pooled to increase the power of the point estimates.

#### Risk of bias assessment

A modified Cochrane risk of bias assessment tool will be used to assess the quality of studies [31] (Additional file 6). This is an assessment tool that considers the various sources of biases by examining the methods of randomisation, concealment, blinding and handling of missing data. Response for each criteria will either be yes, no or unclear following the predefined guidelines as outlined in Additional file 6.

- Was the randomization procedure adequate?
- Were there more than 100 subjects in each treatment group?
- Was the treatment allocation adequately concealed?
- Were physicians blinded to the intervention?
- Were patients blinded to the intervention?
- Were outcome assessors blinded to the intervention?
- Was incomplete outcome data adequately assessed?
- Was intention-to-treat analysis used?
- Were the treatment and control group similar at baseline?
- Are all pre-specified outcomes of interest reported in the pre-specified way?

Wherever a published protocol for the included study is available, it will be used as a supplementary source of information to enhance the quality assessment of the trial protocol. Again, the assessment will be performed by one reviewer with a second review performing random validation in a sample of the included studies. Quality criteria will be used for extended subgroup analysis.

#### Data synthesis

ITT and adjusted results will be extracted whenever possible. If final scores and change from baseline score are both reported, preference will be given to the final score. For data that needs to be extracted visually from graphs, the readings will be rounded to the nearest 0.5.

Random effects model will be used for all analyses. If there are discrepancies between the direct estimates from MA and indirect estimates from NMA—if the 95 % confidence interval of the difference between the direct and indirect does not cross 0—we will consider the network inconsistent. Further analysis will be performed to identify the reason. However, the presence or absence of incoherence will not be determined solely on statistical method since this method is also subjected to statistical errors. Type I error (falsely assuming that there is direct and indirect evidence are inconsistency) can occur in a complex network because multiple tests have to be performed. Type II error (falsely assuming that the direct and indirect evidence are consistent) can happen when the dataset in the network is small [21]. Hence, the statistical significance of inconsistency will be weigh against its clinical significance.

Other than assessing the discrepancy of the estimates, the NMA will also be able to assess the strength and diversity of the treatment network. Comparisons that are supported by a large number of RCTs can be distinguished from those that are informed by only a small number of RCTs.

One major advantage of using Bayesian approach in NMA is that it is able to produce a result for all comparisons in a connected network without the presence of a common comparator. But on the other hand, different prior distributions can be used which can generate different results, and therefore, a sensitivity analysis is always required. As prior knowledge on exercise efficacy is inconclusive, a non-informative prior will be used in our analysis. Posterior distributions of the model parameters will be utilised to present the results of the NMA.

In IPD predictive regression modelling, only clinically meaningful treatment and covariate interaction terms at study and patient level will be investigated [32]. If sample size is deemed sufficient, different predictive model may be explored for different subgroups. To minimise loss of data, we will avoid dichotomizing continuous predictors for analysis [33]—checks for linearity of predictor-outcome relationship will be performed during modelling. If there is a need to convert continuous predictor to categorical nature, this will be performed prior to data analysis. Multiple imputations may be used to address missing data.

#### Subgroup analysis

Extended subgroup analysis will be performed based on the quality criteria of the study (sample size, blinding etc.), patient characteristics (age, BMI, gender etc.), disease (severity, joint involved etc.), exercise and comparator types. Other subgroup analyses will also be undertaken as appropriate.

#### Sensitivity analysis

Sensitivity analysis will be performed to ascertain if the results are robust. Situations where sensitivity analysis will be indicated include (i) imputations of missing data has been employed; (ii) when some arbitrary decisions have been made with respect to study selection, subgroup data aggregation/segregation; and (iii) when outlying studies are suspected. Small-study effect will be specifically assessed as smaller studies tend to report larger effect size and distort the summary estimates of meta-analysis [34].

#### Meta-regression

Meta-regression will be used to assess the association of various covariates in the model. This includes adjusting for study level covariates such as mean age, gender ratio, sample size, allocation concealment, blinding, duration of treatment and type of exercise. Baseline severity of the disease such as pain score may be included as a covariate when the post-treatment score has been used for analysis in lieu of change score.

## Discussion

Exercise is the cornerstone of treatment for patients with lower-limb osteoarthritis. The results of this study will provide evidence on relative efficacy of different types of exercises in OA. It will also provide evidence on which patient subgroup responds better to what type of exercise. This information will help guide clinical practice to optimise exercise therapy in people with OA.

There are several caveats for this study. Firstly, classification of exercise will be based on whatever the included trials have reported. Exercise is a complex intervention, and there is often overlap between the specific elements involved in each exercise intervention. For example, strengthening exercises often progress to involve an aerobic component and aerobic exercise involving lower limb activity may result in strengthening of muscles relevant to knee OA. Therefore, it is very difficult to have a clear cut difference between different types of exercises.

Secondly, this is not a direct head to head comparative study. The relative efficacy between exercises will be estimated from a common comparator indirectly using a network meta-analysis. Variations between studies would affect the estimate. We therefore aim to use Bayesian statistics to increase the precision of the estimate. Even so, we cannot guarantee that the relative efficacy of the difference between exercises is a 100 % true value. Further head to head comparison may still be needed to confirm the results.

Thirdly, heterogeneity is an inherent problem in meta-analysis because of the diversity in clinical and methodological characteristics. These variations include different study time point, different grade of severity and duration of intervention. Therefore, we will focus on identifying the reason for the heterogeneity by performing sensitivity analyses and subgroup analyses. By doing this, we will be grouping studies that are more homogenous together to synthesise a more precise summary of effect.

Another approach to assess the impact of heterogeneity is through the use of IPD which provides the means to subgroup patients according to their characteristics. Some of these subgroup characteristics may be the source of heterogeneity that can be controlled at the IPD level. IPD can also overcome the limitations of study-level meta-regression where individual characteristics are all averaged. However, IPD has some common limitations. It only provides some but not all individual characteristics for subgrouping, and these characteristics often vary from one trial to another. As a result, only limited information can be obtained from an IPD analysis [31]. Furthermore, getting the IPD data from published trials is extremely difficult. The response rate is often low; hence, the selection bias may be introduced when only selective data are made available for the IPD analysis. We will assess the impact of this bias on the estimate by using aggregate data as substitute in both 1-stage and 2-stage analyses for studies without IPD. Lastly, for both NMA and IPD, our search strategy is relying heavily on electronic resources; publication bias may not be avoided. We will therefore estimate this bias in the analysis.

## Additional files

Additional file 1: PRISMA-P checklist. (DOCX 35 kb) Additional file 2: Table of eligible pairwise comparison. (DOCX 14 kb) Additional file 3: MEDLINE search strategy. (DOCX 16 kb) Additional file 4: Equations for calculations. (DOCX 31 kb) Additional file 5: Abridged data extraction form. (DOCX 75 kb) Additional file 6: Quality assessment form. (DOCX 55 kb)

## Abbreviations

ACSM: American College of Sports Medicine
AMED: Allied and Complementary Medicine Database
BMI: Body mass index
CENTRAL: The Cochrane Central Register of Controlled Trials
CINAHL: Cumulative Index to Nursing and Allied Health Literature
EMBASE: Excerpta Medica Database
EQ: EuroQol
IPD: Individual patient/participant data
ITT: Intention-to-treat
MA: Meta-analysis
MCID: Minimal clinically important difference
NMA: Network meta-analysis
OA: Osteoarthritis
PEDro: Physiotherapy Evidence Database
QoL: Quality of life
RCT: Randomised controlled trials
ROM: Range of motion
SF: Short form
WOMAC: Western Ontario and McMaster Universities Osteoarthritis Index
