# Supplementary material for: Relative efficacy of different types of exercise for treatment of knee and hip osteoarthritis: protocol for network meta-analysis of randomised controlled trials
Source: Syst Rev. 2016 Sep 2;5(1):147. doi: 10.1186/s13643-016-0321-6 (PMC5010721; doi:10.1186/s13643-016-0321-6)
Supplement: Additional file 1: — PRISMA-P checklist. (DOCX 35 kb) [file 13643_2016_321_MOESM1_ESM.docx]

**Equations for effect size calculations***

| **Calculations** | **Reference** | **Note** | |
| --- | --- | --- | --- |
| 1. *Calculation for summary data* 2. When combining groups    - $M_{Gp} =\frac{M_{E}N_{E}+M_{c}N_{C}}{N_{E}+N_{C}}$    - ${SD}_{Gp}=\sqrt{\frac{\left( N_{E}-1 \right){{SD}_{E}}^{2}+\left( N_{C}-1 \right){{SD}_{C}}^{2}+\frac{N_{E}N_{C}}{N_{E}+N_{C}}({M_{E}}^{2}+{M_{C}}^{2}-2M_{E}M_{C})}{N_{E}+N_{C}-1}}$ | [[1](#_ENREF_1)] | M  SD  N  Gp  E  C  SE  UpCI  LwCI  a  b  m  q_3_  q_1_  η (N)    Post  Pre  V | = mean  = standard deviation  = sample size  = new group  = exercise group;  = comparator group  = Standard error of means  = upper confidence interval  = lower confidence interval  = minimum value  = maximum value  = median  = third quartile  = first quartile  = function of N (value provided by Wan et al.)  = post-treatment score  = pre-treatment score  = variance |
| 1. When calculating SD from other summary data for exercise or control group    - $SD=SE\times\sqrt{N}$    - $SD= \frac{(UpCI-LwCI)\times\sqrt{N}}{3.92}$ | [[1](#_ENREF_1)] |  |  |
| 1. When estimating SD from range  - $SD\approx\frac{1}{\sqrt{12}}{[\left( b-a \right)^{2}+ \frac{\left( a-2m+b \right)^{2}}{4}]}^{\frac{1}{2}}$ N≤15 - $SD\approx\frac{b-a}{4}$ 15 < N≤70 - $SD\approx\frac{b-a}{6}$ N>70 | [[2](#_ENREF_2)] |  |  |
| 1. When estimating SD from interquartile range  - $SD\approx\frac{q_{3}-q_{1}}{\eta(N)}$ 5≤N< 200 | [[2](#_ENREF_2)] |  |  |
| 1. *Calculation of effect sizes* |  |  |  |
| 1. Cohen’s *d* from post treatment scores    - $d= \frac{M_{post,E}-M_{post,C}}{{SD}_{post, pooled}}$   Where, ${SD}_{pooled}= \sqrt{\frac{(N_{E}-1){{SD}_{E}}^{2}+(N_{C}-1){{SD}_{C}}^{2}}{N_{E}+N_{C}-2}}$ | [[3](#_ENREF_3)] |  |  |
| 1. Cohen’s *d* from change scores    - $d=\frac{(M_{post,E}-M_{pre,E})}{{SD}_{change,E}}-\frac{(M_{post,C}-M_{pre,C})}{{SD}_{change,C}}$ | [[4](#_ENREF_4)] |  |  |
| 1. Hedge’s *g*    - *J* x *d*   Where,  $J=1-\frac{3}{4\left( N_{E}+N_{C} \right)-9}$ | [[3](#_ENREF_3)] |  |  |
| 1. Variance *g*  - $V_{g}=J^{2}\times V_{d}$   Where,  $V_{d}=\frac{N_{E}+N_{C}}{N_{E}N_{C}}+\frac{d^{2}}{2(N_{E}+N_{c})}$ | [[3](#_ENREF_3)] |  |  |
| 1. Standard error of *g*  - ${SE}_{g}=\sqrt{V_{g}}$ | [[3](#_ENREF_3)] |  |  |

*for study designs with two independent groups (only equations relevant for means are listed since this is expected to be the main form of data encountered)

References:

1. Higgins JPT. Green S. Cochrane Handbook for Systematic Reviews of Interventions Version 5.1.0 [updated March 2011] The Cochrane Collaboration. Available from www. cochrane-handbook, org.

2. Wan X, Wang W, Liu J, Tong T.Estimating the sample mean and standard deviation from the sample size, median, range and/or interquartile range. BMC Med Res Methodol. 2014; 14(1):1-13.

3. Borenstein M, Hedges L V, Higgins JPT and Rothstein HR. Introduction to Meta-Analysis, . Chichester, UK. : John Wiley & Sons Ltd.; 2009.

4. Morris SB, DeShon RP. Combining effect size estimates in meta-analysis with repeated measures and independent-groups designs. Psychol Methods. 2002;7(1):105-125.
