# Supplementary material for: Relative efficacy of different types of exercise for treatment of knee and hip osteoarthritis: protocol for network meta-analysis of randomised controlled trials
Source: Syst Rev. 2016 Sep 2;5(1):147. doi: 10.1186/s13643-016-0321-6 (PMC5010721; doi:10.1186/s13643-016-0321-6)
Supplement: Additional file 2: — Table of eligible pairwise comparison. (DOCX 14 kb) [file 13643_2016_321_MOESM2_ESM.docx]

Table S1

Model of pairwise comparisons that will be used to determine study eligibility

| Pairwise comparison that can be derived from the study | | Study eligibility |
| --- | --- | --- |
| Group 1 | Group 2 |  |
| Exercise A  Exercise A  Exercise A + Intervention X | Exercise B  Non-exercise controls  Intervention X | Yes  Yes  Yes |
| Exercise A  Exercise A | Exercise A+ Intervention X  Doses of exercise A* | No  No |

*such as different mode, intensity, delivery methods or setting of exercise A
