# Supplementary material for: Relative efficacy of different types of exercise for treatment of knee and hip osteoarthritis: protocol for network meta-analysis of randomised controlled trials
Source: Syst Rev. 2016 Sep 2;5(1):147. doi: 10.1186/s13643-016-0321-6 (PMC5010721; doi:10.1186/s13643-016-0321-6)
Supplement: Additional file 3: — MEDLINE search strategy. (DOCX 16 kb) [file 13643_2016_321_MOESM3_ESM.docx]

**Medline Search Strategy**

1. exercise/ or physical conditioning, human/
2. Circuit-Based Exercise/
3. exercise therapy/ or motion therapy, continuous passive/ or muscle stretching exercises/ or plyometric exercise/ or resistance training/ or hydrotherapy/ or rehabilitation/ or "activities of daily living"/ or dance therapy/
4. muscle strength/ or physical endurance/ or anaerobic threshold/ or exercise tolerance/ or physical fitness/ or postural balance/ or posture/ or psychomotor performance/ or "range of motion, articular"/
5. Pliability/
6. movement/ or motor activity/ or exercise/
7. Physical Exertion/
8. Mind-Body Therapies/
9. running/ or jogging/ or swimming/ or walking/
10. Isometric Contraction/
11. exercise movement techniques/ or breathing exercises/ or qigong/ or tai ji/ or yoga/ or pilates
12. propriocepti$.ab,ti.
13. balanc$.ab,ti.
14. aqua$.ab,ti.
15. cycl$.ab,ti.
16. aerobic.ab,ti.
17. strength$.ab,ti.
18. (tai-ji or taiji or taijiquan or tai ji quan or tai chi or taichi or t ai chi or t'ai chi or tai chi chuan).ab,ti.
19. (qigong or qi gong or chi kung or chikung or ch i kung or ch'i kung).ab,ti.
20. therap$.ab,ti.
21. physiotherap$.ab,ti.
22. train$.ab,ti.
23. neuromuscular training.ab,ti.
24. treadmill.ab,ti.
25. 1 or 2 or 3 or 4 or 5 or 6 or 7 or 8 or 9 or 10 or 11 or 12 or 13 or 14 or 15 or 16 or 17 or 18 or 19 or 20 or 21 or 22 or 23 or 24
26. Osteoarthritis, Hip/ or Osteoarthritis, Knee/
27. coxarthritis.mp.
28. coxarthr$.ab,ti.
29. gonarthr$.ab,ti.
30. (knee$ adj3 pain).ab,ti.
31. (hip adj3 pain).ab,ti.
32. osteoarthr$.ab,ti.
33. degenerative joint disease?.ab,ti.
34. osteoarthritis/
35. (Osteoarthriti$ or OA or osteo arthriti$ or osteoarthros$ or osteo arthros$ or arthropath$ or arthrosis or arthroses).ti,ab.
36. Hip Joint/
37. Hip/
38. hip$.ab,ti.
39. Knee/
40. knee$.ab,ti.
41. knee joint/ or patellofemoral joint/
42. 26 or 27 or 28 or 29 or 30 or 31
43. 32 or 33 or 34 or 35
44. 36 or 37 or 38 or 39 or 40 or 41
45. 43 and 44
46. 42 or 45
47. randomized controlled trial.pt.
48. controlled clinical trial.pt.
49. (placebo or (standard adj3 care)).ab.
50. clinical trials as topic.sh.
51. random$.ab,ti.
52. trial$.ab,ti.
53. RCT.ab,ti.
54. 47 or 48 or 49 or 50 or 51 or 52 or 53
55. limit 54 to humans
56. 25 and 46 and 55
