# Supplementary material for: Relative efficacy of different types of exercise for treatment of knee and hip osteoarthritis: protocol for network meta-analysis of randomised controlled trials
Source: Syst Rev. 2016 Sep 2;5(1):147. doi: 10.1186/s13643-016-0321-6 (PMC5010721; doi:10.1186/s13643-016-0321-6)
Supplement: Additional file 5: — Abridged data extraction form. (DOCX 75 kb) [file 13643_2016_321_MOESM5_ESM.docx]

**DATA EXTRACTION FORM_**

**Date of review :** enter a date. **ID of reviewer :** enter initials

**Title of article :**  enter article title.

**Author(s) :** first 3 authors.

1. **Study characteristics**

| **Country:** Country where study was done. **Year:** State study date | | | |
| --- | --- | --- | --- |
| **Setting:** Group  Individual  Home based  Unclear  Others: | | | |
| **Definition of OA used: Self-reported**  **Radiographic**  **Symptomatic**  **ACR**  **Unclear  Others:** Click here to enter text. | | | |
| **Grouping:**   \| Control \| Intervention 1 \| Intervention 2 \| \| --- \| --- \| --- \| \| Detail description:  Enter description of exercise details \| Body  Mind  Mixed  Unclear  Detail description:  Enter description of exercise details \| Body  Mind  Mixed  Unclear  Detail description:  Enter description of exercise details \| | | | |
| **Design:** Parallel  Crossover  Others: Other types | | | |
| **Follow-up duration:**  Time point 1 - First ; Time point 2 - Second ; Time point 3 – Third | | | |
| **Intervention duration:** **wks** | | | |
| **Outcome:** | | | |
|  | Primary 1 -  text. | Primary 2 - text. |  |
|  | Secondary 1 - text | Secondary 2 - text. | Secondary 3 - text |

1. **Participants Characteristics**

| **Data based on: per protocol**  **ITT analysis**  **Types of OA: Knee**  **Hip** | | | | |
| --- | --- | --- | --- | --- |
| **Demographic** | **Control** | **Intervention 1** | **Intervention 2** | **Total** |
| **Number of participants** |  |  |  |  |
| **Age (mean ± SD)** |  |  |  |  |
| **Male/ Female** |  |  |  |  |
| **Knee/Hip ( if mixed cohort)** |  |  |  |  |
| **Severity/grading of OA** |  |  |  |  |
| **Duration of OA (mean ± SD)** |  |  |  |  |
| **Attrition (number)** |  |  |  |  |
| **Adherence of exercise (if relevant)** |  |  |  |  |

1. **Outcome**

| **Outcome:** Primary 1. **Adjusted:** | | | | | | | | | |
| --- | --- | --- | --- | --- | --- | --- | --- | --- | --- |
|  | **Control** | | | **Intervention 1** | | | **Intervention 2** | | |
|  | Mean/ proportion | SD | 95% CI | Mean/ proportion | SD | 95% CI | Mean/ proportion | SD | 95% CI |
| **Baseline** |  |  |  |  |  |  |  |  |  |
| text. |  |  |  |  |  |  |  |  |  |
| text. |  |  |  |  |  |  |  |  |  |
| text. |  |  |  |  |  |  |  |  |  |
| **Outcome:** Primary 2 **Adjusted:** | | | | | | | | | |
|  | **Control** | | | **Intervention 1** | | | **Intervention 2** | | |
|  | Mean/ proportion | SD | 95% CI | Mean/ proportion | SD | 95% CI | Mean/ proportion | SD | 95% CI |
| **Baseline** |  |  |  |  |  |  |  |  |  |
| text. |  |  |  |  |  |  |  |  |  |
| text. |  |  |  |  |  |  |  |  |  |
| text. |  |  |  |  |  |  |  |  |  |
| **Outcome:**Secondary 1 **Adjusted:** | | | | | | | | | |
|  | **Control** | | | **Intervention 1** | | | **Intervention 2** | | |
|  | Mean/ proportion | SD | 95% CI | Mean/ proportion | SD | 95% CI | Mean/ proportion | SD | 95% CI |
| **Baseline** |  |  |  |  |  |  |  |  |  |
| text. |  |  |  |  |  |  |  |  |  |
| text. |  |  |  |  |  |  |  |  |  |
| text. |  |  |  |  |  |  |  |  |  |

| **Outcome:** Secondary 2. **Adjusted:** | | | | | | | | | |
| --- | --- | --- | --- | --- | --- | --- | --- | --- | --- |
|  | **Control** | | | **Intervention 1** | | | **Intervention 2** | | |
|  | Mean/ proportion | SD | 95% CI | Mean/ proportion | SD | 95% CI | Mean/ proportion | SD | 95% CI |
| **Baseline** |  |  |  |  |  |  |  |  |  |
| text. |  |  |  |  |  |  |  |  |  |
| text. |  |  |  |  |  |  |  |  |  |
| text. |  |  |  |  |  |  |  |  |  |

| **Outcome:** Secondary 3. **Adjusted:** | | | | | | | | | |
| --- | --- | --- | --- | --- | --- | --- | --- | --- | --- |
|  | **Control** | | | **Intervention 1** | | | **Intervention 2** | | |
|  | Mean/ proportion | SD | 95% CI | Mean/ proportion | SD | 95% CI | Mean/ proportion | SD | 95% CI |
| **Baseline** |  |  |  |  |  |  |  |  |  |
| text. |  |  |  |  |  |  |  |  |  |
| text. |  |  |  |  |  |  |  |  |  |
| text. |  |  |  |  |  |  |  |  |  |

**Comments:**

*The following unformatted table is provided to allow documentation of additional information e.g. additional treatment groups or treatment outcomes.

| **Outcome: Adjusted:** | | | | | | | | | |
| --- | --- | --- | --- | --- | --- | --- | --- | --- | --- |
|  |  | | |  | | |  | | |
|  | **Mean/ proportion** | **SD** | **95% CI** | **Mean/ proportion** | **SD** | **95% CI** | **Mean/ proportion** | **SD** | **95% CI** |
| **Baseline** |  |  |  |  |  |  |  |  |  |
| text. |  |  |  |  |  |  |  |  |  |
| text. |  |  |  |  |  |  |  |  |  |
| text. |  |  |  |  |  |  |  |  |  |
