# Supplementary material for: Relative efficacy of different types of exercise for treatment of knee and hip osteoarthritis: protocol for network meta-analysis of randomised controlled trials
Source: Syst Rev. 2016 Sep 2;5(1):147. doi: 10.1186/s13643-016-0321-6 (PMC5010721; doi:10.1186/s13643-016-0321-6)
Supplement: Additional file 6: — Quality assessment form. (DOCX 55 kb) [file 13643_2016_321_MOESM6_ESM.docx]

**QUALITY ASSESSMENT FORM_**

**Date of review :** enter a date. **ID of reviewer :** enter initials

**Title of article :**  enter article title. Comments: enter any comments

**Author(s) :** first 3 authors.

| *Please see Appendix 1 for criteria* | Yes | No | Unclear |
| --- | --- | --- | --- |
| 1. Was the randomisation procedure adequate?   Comments: |  |  |  |
| 1. Were there more than 100 subjects in each treatment group?   Comments: |  |  |  |
| 1. Was the treatment allocation adequately concealed?   Comments: |  |  |  |
| 1. Were physicians blinded to the intervention?   Comments: |  |  |  |
| 1. Were patients blinded to the intervention?   Comments: |  |  |  |
| 1. Were outcome assessors blinded to the intervention?   Comments: |  |  |  |
| 1. Was incomplete outcome data adequately assessed?   Comments: |  |  |  |
| 1. Was intention-to-treat analysis used?   Comments: |  |  |  |
| 1. Were the treatment and control group similar at baseline?   Comments: |  |  |  |
| 1. Are all pre-specified outcomes of interest reported in the pre-specified way? |  |  |  |

***Criteria for risk of bias assessment***

| Criteria | Yes | No |
| --- | --- | --- |
| Was the randomisation procedure adequate? | A random component in the sequence generation process is used, such as:   - Using to a random number table - Using a computer random number generator - Tossing a coin - Shuffling cards or envelopes - Throwing dice - Drawing lots - Minimisation | A non-random component is used in the allocation process, such as:   - Sequence generated using odd or even date of birth - Sequence based on a rule based on date of admission or hospital number - Allocation decision is made by clinician or patient - Allocation is based on clinical or laboratory test findings |
| Were there more than 100 patients in each treatment group? | More than 100 patients were randomized in each group | Less than 100 patients were randomized to each group |
| Was the treatment allocation adequately concealed? | Patients and physicians could not foresee allocation assignment, because an adequate method was used to conceal allocation. Adequate allocation concealment methods include:   - Central allocation - Opaque, sealed, sequentially numbered assignment envelopes are used | Patients or physicians could potentially foresee allocation. Inadequate allocation concealment procedures include:   - The use of an open random allocation schedule - Assignment envelopes are not appropriate, such that envelopes may be unsealed, not sequentially numbered, or see-through - Rotation or alternation - Methods based on date of birth, case record number, or other patient identifiers |
| Were physicians blinded to the intervention? | Physicians were adequately blinded and it is unlikely that the blinding could be broken | No blinding, incomplete blinding, or blinding attempted but likely to have been broken |
| Were patients blinded to the intervention? | Patients were adequately blinded and it is unlikely that the blinding could be broken | No blinding, incomplete blinding, or blinding attempted but likely to have been broken |
| Were outcome assessors blinded to the intervention? | Treatment choice is not evident when measuring outcome.   - If outcome is patient-reported, then the answer for “Were patients blinded to the intervention?” will be the same for this question. - For physician-assessed outcomes, this is dependent on whether the treatment allocation can be identified from examination of the patient or their tests | Treatment choice is likely to be evident when measuring outcome   - If outcome is patient-reported, then the answer for “Were patients blinded to the intervention?” will be the same for this question. - For physician-assessed outcomes, this is dependent on whether the treatment allocation can be identified from examination of the patients or their tests |
| Was incomplete outcome data adequately assessed? | YES – ≥2 of the following questions as “yes”   - Is there any missing outcome data? - Are the reasons for missing data unlikely to be related to the outcome? - Has missing data been imputed using appropriate methods, such as multiple imputations | NO – < 2 of the following questions as “yes”   - Is there any missing outcome data? - Are the reasons for missing data unlikely to be related to the outcome? - Has missing data been imputed using appropriate methods, such as multiple imputations |
| Was intention-to-treat analysis used? | - All randomised patients are analysed according to the group they were allocated to, regardless of non-compliance - No patients were lost throughout the trial - The analysis has been characterised by the authors | - Per-protocol analysis is used to analyse only patients that adhered fully to their allocated treatment, - Patients who dropped-out or moved between treatment groups were excluded from analysis |
| Were the treatment and control group similar at baseline? | Treatment and control group were similar regarding patient demographics and pain parameters | Treatment and control groups were dissimilar with regards to patient demographics and pain parameters |
| Are all pre-specified outcomes of interest reported in the pre-specified way? | All the study’s pre-specified outcomes of interest are reported in the pre-specified way | Outcome measures were not pre-specified, such as:   - Not all the pre-specified outcomes have been reported - Outcomes are not reported using the measurements or methods pre-specified - Outcomes reported had not been pre-specified - Outcomes of interest have been incompletely reported - The report does not report outcomes that would be expected from such a study |
